# Effect of Neuromuscular Training Program on Quality of Life After COVID-19 Lockdown Among Young Healthy Participants: A Randomized Controlled Trial

**DOI:** 10.3389/fpsyg.2022.844678

**Published:** 2022-04-12

**Authors:** Dragan Marinkovic, Drazenka Macak, Dejan M. Madic, Goran Sporis, Dalija Kuvacic, Dajana Jasic, Vilko Petric, Marijan Spehnjak, Aleksandra Projovic, Zoran Gojkovic

**Affiliations:** ^1^Faculty of Sport and Physical Education, University of Novi Sad, Novi Sad, Serbia; ^2^Faculty of Kinesiology, University of Zagreb, Zagreb, Croatia; ^3^Department of Economics, University of Applied Sciences Zagreb, Zagreb, Croatia; ^4^Department of Teachers’ and Preschool Teachers’ Education, University of Zadar, Zadar, Croatia; ^5^Faculty of Teacher Education, University of Rijeka, Rijeka, Croatia; ^6^Archdiocese of Zagreb, Zagreb, Croatia; ^7^Primary School “Stefan Nemanja”, Niš, Serbia; ^8^Government of the Autonomous Province of Vojvodina, Provincial Secretariat for Health Care, Novi Sad, Serbia; ^9^Faculty of Medicine, University of Novi Sad, Novi Sad, Serbia

**Keywords:** COVID-19, exercise, well-being, neuromuscular training, quality of life

## Abstract

Study in the period of coronavirus disease 2019 (COVID-19) lockdown and the effect of different exercise training programs on the quality of life (QoL) dimension are limited. This randomized control study as a part of which the impact of an 8-week neuromuscular training program on the 90 healthy young individuals’ QoL after COVID-19 lockdown was assessed using a short form of the WHOQOL-BREF questionnaire comprising of four domains (physical health, psychological health, social relations, and the environment). The intervention group (NT) (*n* = 47) took part in a neuromuscular training program consisting of dynamic neuromuscular stabilization and whole-body vibration training. In contrast, the control group (CG) (*n* = 43) did not participate in any programmed physical activity. From pre- to post-intervention test, the NT group significantly and substantially improved [mean change (95% CI)] all the QoL domains, physical for 12.78 scores (8.89, 16.64), psychological for 13.12 scores (9.51, 16.74), social relationships for 20.57 scores (16.12, 25.02), and environmental for 24.40 scores (21.45, 27.35). These results suggest that the NT program could enhance QoL in young and healthy participants following COVID-19 lockdown.

## Introduction

In an effort to curb coronavirus disease 2019 (COVID-19) spread, governments across the world have adopted different public health policies (Nussbaumer-Streit et al., 2020), some of which have had an adverse impact on mental health and quality of life (QoL) (Battaglia et al., 2016; Ku et al., 2016; Bernard et al., 2018; Delle Fave et al., 2018; Nawrocka and Polechoński, 2019; Brooks et al., 2020). Extant study on mental health in the context of COVID-19 epidemic and government-imposed restrictions on movement and social contact indicate that such policies have had a detrimental effect on emotional and social functioning (Pfefferbaum and North, 2020) and mental health (Gunnell et al., 2020; Hossain et al., 2020), thus increasing the incidence of depression, anxiety, post-traumatic stress, anger, and confusion (Pieh et al., 2020), and risk of psychosocial strain (Ammar et al., 2021). Pandemics are also linked to a variety of sociopsychosocial stresses induced by quarantine and significant changes in daily routine, such as the limited potential for physical activity (PA) (Ammar et al., 2020; Carriedo et al., 2020; Maugeri et al., 2020; Pišot et al., 2020; Trabelsi et al., 2021; Schoofs et al., 2022). As during lockdown, PA levels decreased considerably in the most population groups (among young people in particular), the resulting lifestyle that exacerbated these mental and physical health issues (Yanovski et al., 2000; Altena et al., 2020; Clay and Parker, 2020; Jiménez-Pavón et al., 2020). COVID-19 confinement has made it difficult to comply with the WHO guidelines stipulating 150 min of moderate-to-mild PA per week or 75 min of intensive PA per week (World Health Organization, 2020). Consequently, given the well-established link between a low-level PA lifestyle and mental health and, thus, QoL, it is important to investigate if these adverse impacts can be reversed by a short-term training program.

Previous studies on this topic indicate that exercise and physical training have a significant positive impact on QoL (Spirduso and Cronin, 2001; de Vreede et al., 2007). Similarly, ample body of evidence confirms that different depression symptoms are inversely correlated with physical fitness (Galper et al., 2006; Tolmunen et al., 2006; Valtonen et al., 2009). More recently, Becofsky et al. (2015) reported that lack of fitness is more strongly linked to the risk of depression than being overweight.

Indirect and direct benefits of PA and exercise for physical health and overall well-being are well-known (Stathi et al., 2002; Lehnert et al., 2012). Nonetheless, in the last few decades, there has been a growing interest in the effects of exercise or PA interventions on mental health and QoL dimensions (Becofsky et al., 2015). The results obtained by Khosravi (2020) suggest that regular PA can help to minimize the risk of developing mental health issues (namely, depression, anxiety, and stress), while findings published by Sui et al. (2009) and Kandola et al. (2019) indicate that being physically active might help to avoid symptoms of depression. Following a randomized controlled experiment, Martin et al. (2009) similarly concluded that there was a substantial and positive association between the quantity of PA and improvements in physical and mental health measured *via* QoL parameters. When the benefits of a 24-week resistance training program were investigated by McLafferty et al. (2004), most participants reported significant improvements in overall mood and marked reduction in anger, confusion, and mental tension, after completing the experimental program. More recently, it was reported that the intervention group was more satisfied with their health and QoL compared to the controls with improvement in body weight and body mass index (BMI) and the authors posited that these benefits of exercise could have contributed to QoL improvements (Colak and Baskan, 2020). Askari et al. (2020) adopted aerobic exercise intervention with the multidimensional approach in addition to normal care and found that this intervention was beneficial in alleviating different categories of depression (physical, emotional, and cognitive), while improving the participants’ psychological health and social interactions aspects of QoL. Several researchers have also explored the link between exercise intensity, type of exercise, and QoL domains. Low-intensity jogging, cycling, aerobic exercises, swimming, walking, and dancing have been shown to diminish depression and anxiety symptoms (Guszkowska, 2004). Stracciolini et al. (2020) and Su et al. (2021) similarly noted that participation in different sports activities can improve mental health and anxiety QoL scores. Moreover, findings from Slimani et al. (2020) study yielded that low-intensity PA can also help minimize the negative psychological effects of isolation. West et al. (2004) and Nguyen et al. (2021) concurred with this view and proposed alternative PA programs like Hatha yoga, unsupervised exercise, and African dancing as a means of improving psychological and mental health, suggesting that such activities should be promoted to enhance QoL.

As most of the aforementioned studies were conducted on samples drawn from the general population, it is important to investigate the link between PA and QoL in young and healthy individuals. As to the best of the authors’ knowledge, the effects of complex neuromuscular training (NT) in this cohort have never been investigated, especially in the context of COVID-19 lockdown, these gaps are addressed in this study. Therefore, this study aimed to determine the effects of 8-week NT on four QoL dimensions in a group of untrained healthy young individuals after COVID-19 lockdown with the hypothesis that an 8-week intervention consisting of three NT exercise sessions per week should result in significant increases in psychological dimensions and QoL.

## Materials and Methods

### Study Design and Procedures

This randomized control study evaluated the effects of an 8-week experimental training program on quality of life of faculty of sport and physical education students at the University of Novi Sad, Serbia. The initial and final evaluations were performed on the 2 days preceding and following the 2 months intervention period, respectively. Participants performed a 2-months neuromuscular training (NT) intervention program with dynamic neuromuscular stabilization (DNS) protocol and whole-body vibration training (WBVT), while the control group (CG) did not exercise or use any training intervention or other habitual training during 8 weeks. Experimental programs were valid when the participants finished at least 80% of all the training sessions. To guarantee the quality and correct execution of training protocols, professional coaches and researchers supervised all the training programs.

### Participants

**FIGURE 1:**
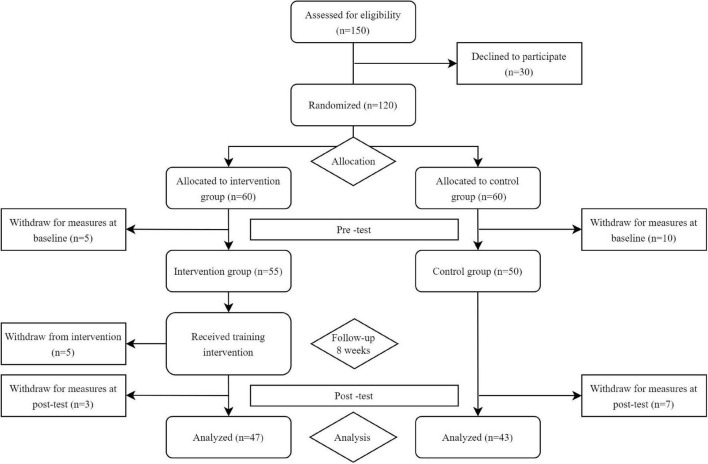
Flow diagram of participant enrollment, allocation, and analysis.

A gender-balanced group of 90 healthy young participants (age 24.02 ± 2.07 years; height 174.98 ± 8.98 cm; and weight 68.16 ± 12.28 kg) was enrolled in this study. Exclusion criteria were: (i) history of neurological or musculoskeletal disorders; (ii) clinical conditions that could impair balance; (iii) a regular PA practice during lockdown; and (iv) using drugs, alcohol, and other substances. The study sample was randomly divided into the NT group (*n* = 47) and the CG group (*n* = 43). At baseline no significant differences (*p* > 0.05) were found between the groups in age, height, weight, and BMI (Table 1). Each subject, after explanation of the experimental protocol, provided a written informed consent before participating in this study, in accordance with the Declaration of Helsinki and approved by the Novi Sad University Human Research Ethics Committee (ethical approval number: 234/2020). The flow diagram of participants through this study is given in Figure 1.

**TABLE 1:** Characteristics of participants.

Variables	TOTAL (*n* = 90)	NT (*n* = 47)	CG (*n* = 43)
Gender (male/female)	45/45	24/23	21/22
Age(years)	23.94 ± 1.9	23.73 ± 2.05	24.16 ± 1.89
Weight (kg)	66.8 ± 12.0	67.41 ± 12.51	68.17 ± 11.63
Height (cm)	174.9 ± 8.98	174.92 ± 9.44	174.87 ± 8.39
BMI (kg/m^2^)	22.0 ± 2.6	21.85 ± 2.44	22.18 ± 2.87

*Data is presented as AM ± SD. AM, arithmetic mean; SD, standard deviation; NT, neuromuscular training group; CG, Control group.*

### Measures

#### Quality of Life

In this study, QoL was assessed using the short form of the WHOQOL-BREF questionnaire (World Health Organization, 1998) comprising of 26 items pertaining to four domains: physical health, psychological health, social relations, and the environment. Each item was rated by the respondents on a five-point Likert scale (1—Not at all; 2—A little; 3—A moderate amount; 4—Very much; and 5—An extreme amount). As the WHOQOL-BREF reliability and validity have been confirmed in prior study (Kalfoss et al., 2021), it was adopted in this study without further evaluations or modifications.

### Training Intervention

In Serbia, the first case of COVID-19-positive patient was reported on 6 March 2020 and national authorities declared a state of emergency on 15 March 2020 to keep people at home, minimizing physical contacts, namely, restrictions of closing universities, schools, fitness centers, sports facilities, parks, and entertainment centers. In different periods during March, April, and May, restrictions were applied by the official complete ban from leaving homes for all the age groups. According to the national lockdown strategy, most of the restrictions were finished by the end of May 2020 (Vuković et al., 2021) when the research training program was started.

During the 2 months, participants’ training consisted of 3 weekly training sessions. The protocol consisted of a 50-min exercises program with a warm-up and cool-down period per training session. The structural core of training included 20 min of DNS training and a 20-min WBVT. Both protocols were designed in line with the previous research and training recommendations. Exercises in WBVT were performed on Power Plate Next Generation vibration platform (Power Plate North America, Chicago, Illinois, United States). The program consisted of 6–8 exercises (static and dynamic) for balance and postural stability (PS). Exercise progressively increases by the level of difficulty. During the training process, the frequency was also increased in range from 20 to 35 Hz in the last week of the experiment (1.8 Hz increase per week); duration of exercise ranged from 20 to 60 s (5 s increase per week) followed by 1-min seated rest. Resting periods between sets were constant from the start to the end of the training process. Basic principles and the procedures were adapted from previous research (Torvinen et al., 2002; Jordan et al., 2005; Fort et al., 2012; Piecha et al., 2014). WBVT followed by a 20-min period of DNS training including specific movement exercises according to the DNS approach accompanied by breathing exercises and in line with principles from previous studies (Frank et al., 2013; Mahdieh et al., 2020). To ensure that each exercise was executed correctly, participants were supervised by the coaches and the research team. The control group did not participate in any exercise, training, or sport-specific program during the 8-week experiment.

### Data Analysis

G*power 3.1 power analysis software (Heinrich-Heine-University, Düsseldorf, Germany) determined the minimum total sample size (*n* = 50) given the critical *F* = 4.04, an effect size *f* = 0.20 (partial η^2^ = 0.04), *p* = 0.05, 1 – β = 0.80, groups and time points = 2, and correlation among the measurements = 0.50. Data are presented as mean and 95% CIs unless otherwise stated. A *t*-test for independent samples tested whether the baseline study outcomes differed among the groups. A 2 (pretest vs. post-test) × 2(NT vs. CG) mixed ANOVA model evaluated the 8-week effects of neuromuscular training on the QoL domains (physical, psychological, social relationships, and environmental). The Kolmogorov–Smirnov test confirmed normality of residuals, and the Levene’s and Box’s tests accepted the homogeneity of the variances and covariance matrices, respectively. We inspected whether mean changes (95% CI) from initial to final testing in each QoL domain significantly depended on whether subjects completed NT or not using a time*group interaction effect. Given a significant time*group interaction effect, the simple main effects of time followed (*post-hoc*) which tested the significance of mean changes [95% CI] from initial to final testing within the groups with a Bonferroni adjusted *p* values and 95% CIs. Partial eta squared (partial η^2^) is reported as the effect size measure for the interaction effects and classified as small (0.01), moderate (0.06), and large (0.14) (Cohen, 2013). The Hedges’s *g*_av_ with 95% CIs designated the size of simple main effect of time and interpreted as small (± 0.20), moderate (± 0.50), and large (± 0.8). The level of significance was set at *p* ≤ 0.05. All the statistical analyses were performed with the SPSS statistical software (SPSS 23.0, IBM Incorporation, Chicago, Illinois, United States).

## Results

Baseline values of physical (*p* = 0.47), psychological (*p* = 0.47), social relationships (*p* = 0.74), and environmental (*p* = 0.61) QoL domains were similar across the groups (Table 2). Both the groups achieved, on average, similar levels of QoL.

**TABLE 2:** Baseline values of outcomes for neuromuscular training (NT) group (*n* = 47) and control group (CG) (*n* = 43).

Outcomes	NT	CG
	Mean [95% CI]	Mean [95% CI]
Physical QoL (score)	49.16 [46.65, 51.68]	47.92 [45.61, 50.24]
Psychological QoL (score)	51.33 [48.89, 53.78]	50.10 [47.57, 52.62]
Social Relationship QoL (score)	52.31 [48.82, 55.79]	53.10 [49.79, 56.41]
Environmental QoL (score)	49.53 [47.38, 51.68]	50.29 [48.26, 52.32]

**FIGURE 2:**
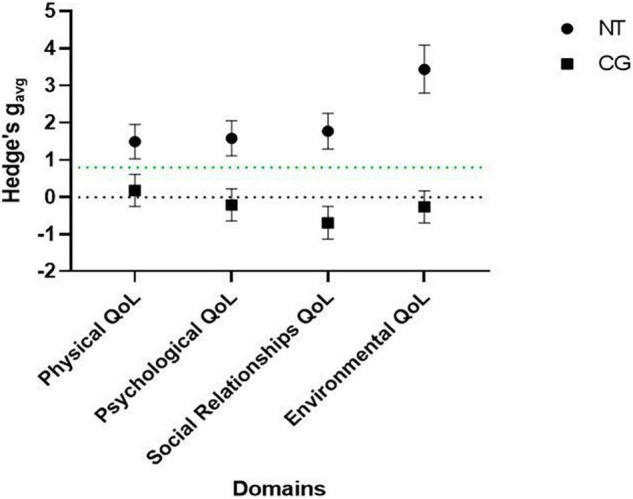
Effect sizes with 95% CIs (Hedges’s gav) for pre-post changes of Physical, Psychological, Social Relationships, Environmental QoL domains in the NT and CG.

The 8-week-mean changes of all the QoL domains significantly differed between the NT group and CG to a large extent [physical: *F*_(1,88)_ = 16.19, *p* < 0.001, partial η^2^ = 0.16; psychological: *F*_(1,88)_ = 32.21, *p* < 0.001, partial η^2^ = 0.27; social relationships: *F*_(1,88)_ = 76.45, *p* < 0.001, partial η^2^ = 0.47; and environmental: *F*_(1,88)_ = 151.57, *p* < 0.001, partial η^2^ = 0.63]. On average, the NT group significantly and substantially improved all QoL domains [mean change (95% CI)], physical for 12.78 scores (8.89, 16.64), psychological for 13.12 scores (9.51, 16.74), social relationships for 20.57 scores (16.12, 25.02), and environmental for 24.40 scores (21.45, 27.35). In CG, no significant mean changes, however, were observed in any QoL domains after 8 weeks (*p* > 0.05), except mean scores for social relationships QoL domain which significantly lowered for −7.75 scores (−12.40, −3.10) to a moderate extent. Figure 2 illustrates the comparison of effect sizes for 8-week-mean changes within the groups and the space above the green line presents large improvements.

## Discussion

The purpose of this study focusing on a group of healthy young individuals was to determine the effects of 8-week NT administered after COVID-19 lockdown on all the four QoL dimensions. Baseline results confirmed previous studies that indicate a low level of mental and psychological domains in people during COVID-19 lockdowns (Bonichini and Tremolada, 2021; Epifanio et al., 2021; Ferreira et al., 2021). Analysis of their results and comparison with the control group revealed that participation in the NT program could lead to QoL enhancement in young healthy participants. These results also concur with the findings reported in extant literature focusing on the link between PA and QoL in the general population (Stracciolini et al., 2020; Su et al., 2021) and specifically during COVID-19 (Slimani et al., 2020).

Dynamic neuromuscular stabilization and vibration training present a multidimensional approach that combines different breathing, core strength, and stabilization exercises with specific exercises performed on a vibration platform. Even though they adopted a different methodology, both Prem et al. (2013) and Montoro et al. (2018) noted that diaphragmatic breathing exercise and vibration training could improve QoL in both the short term and long term. Similarly, Guszkowska (2004) and Slimani et al. (2020) observed that even moderate exercise (as was the case in our study) can lead to marked improvements in QoL.

In this respect, this study provides a modest contribution to the ongoing discussions about the side effects of COVID-19 lockdown on QoL and its specific domains, indicating that NT could lead to improvement in QoL parameters and minimal negative mental health consequences in circumstances similar to the pandemic. Given the well-established link between PA (whether as a part of an exercise program or a generally active lifestyle) and QoL dimensions, namely, physical health (Battaglia et al., 2016; Nawrocka et al., 2019) mental wellness and psychological well-being (Bernard et al., 2018; Trabelsi and Ammar, 2021), environmental domain (Valenti et al., 2008; Ku et al., 2016), and emotions (Delle Fave et al., 2018), it is reasonable to assume that PA would be beneficial following a prolonged period of physical inactivity, as was the case during COVID-19 lockdown. This hypothesis was supported in this study, as an 8-week intervention consisting of just three NT exercise sessions per week resulted in significant increases in psychological dimensions and QoL. Thus, we argue that incorporating NT programs into daily lifestyle would be essential to support mental health in all populations, especially among young people, to combat the adverse effects of lifestyle caused by COVID-19 lockdown or circumstances similar to the pandemic. Furthermore, varied exercise routines should be customized to the participant’s fitness level, and a progressive strategy of intensity and workout volume should be used (Chtourou et al., 2020). In addition, main educational authorities should pay much more attention to providing enough knowledge and improving health literacy in conditions like COVID-19 lockdown (Geets Kesic et al., 2021).

When interpreting these findings, it is important to note some of the study limitations, including the relatively small sample size and the fact that the intervention was conducted after COVID-19 lockdown, during which all participants significantly reduced their PA levels. Nonetheless, as all our research subjects were young and healthy individuals, recruited from the same social setting, it is possible that the same intervention would yield different results in cohorts with different socioeconomic and demographic characteristics. Finally, we acknowledge that, since the participants were not asked to report on their activity levels outside of the study protocol, our results could be influenced by variations in their daily routine. However, those activities were not included organized, systematic, and planned PA. Therefore, including accelerometer, mobile application or wearable sensors in future studies would clarify the intervention-induced effects.

## Conclusion

This study aimed to determine the effects of 8-week NT on four QoL dimensions in a group of untrained healthy young individuals after COVID-19 lockdown. Our findings indicate that, due to the beneficial effects of our intervention on all four QoL domains, the NT and different PAs should be considered for inclusion in public health policy to lessen the adverse effects of lockdown or situation similar to the pandemic.

## Data Availability Statement

The original contributions presented in the study are included in the article/supplementary material, further inquiries can be directed to the corresponding author.

## Ethics Statement

The studies involving human participants were reviewed and approved by Ethics Committee of the Faculty of Sport and Physical Education, University of Novi Sad Human Research Ethics Committee guidelines (ethical approval number: 234/2020). The participants provided their written informed consent to participate in this study.

## Author Contributions

DMar and DMac: conceptualization, editing, and revision. DMac, DK, and DJ: methodology. DMad and GS: validation. DMac: formal analysis. DM and MS: investigation. DM and DK: writing—original draft preparation. ZG, DJ, VP, and AP: writing—review and editing. DM, GS, and DMad: supervision. ZG and DM: revision and correction of the manuscript. All authors have read and agreed to the published version of the manuscript.

## Conflict of Interest

The authors declare that the research was conducted in the absence of any commercial or financial relationships that could be construed as a potential conflict of interest.

## Publisher’s Note

All claims expressed in this article are solely those of the authors and do not necessarily represent those of their affiliated organizations, or those of the publisher, the editors and the reviewers. Any product that may be evaluated in this article, or claim that may be made by its manufacturer, is not guaranteed or endorsed by the publisher.
