# Corrigendum: Effect of neuromuscular training program on quality of life after COVID-19 lockdown among young healthy participants: A randomized controlled trial

**DOI:** 10.3389/fpsyg.2023.1161581

**Published:** 2023-02-28

**Authors:** Dragan Marinkovic, Drazenka Macak, Dejan M. Madic, Goran Sporis, Dalija Kuvacic, Dajana Jasic, Vilko Petric, Marijan Spehnjak, Aleksandra Projovic, Zoran Gojkovic

**Affiliations:** ^1^Faculty of Sport and Physical Education, University of Novi Sad, Novi Sad, Serbia; ^2^Faculty of Kinesiology, University of Zagreb, Zagreb, Croatia; ^3^Department of Economics, University of Applied Sciences Zagreb, Zagreb, Croatia; ^4^Department of Teachers' and Preschool Teachers' Education, University of Zadar, Zadar, Croatia; ^5^Faculty of Teacher Education, University of Rijeka, Rijeka, Croatia; ^6^Archdiocese of Zagreb, Zagreb, Croatia; ^7^Primary School “Stefan Nemanja”, Niš, Serbia; ^8^Government of the Autonomous Province of Vojvodina, Provincial Secretariat for Health Care, Novi Sad, Serbia; ^9^Faculty of Medicine, University of Novi Sad, Novi Sad, Serbia

**Keywords:** COVID-19, exercise, well-being, neuromuscular training, quality of life

In the published article, there was an error regarding the affiliation(s) for Zoran Gojkovic. As well as having affiliation 8, they should also have affiliation 9, “Faculty of Medicine, University of Novi Sad, Novi Sad, Serbia.”

The authors apologize for this error and state that this does not change the scientific conclusions of the article in any way. The original article has been updated.

